# Species composition and distribution data of benthic foraminifera from the straits of malacca during the early holocene

**DOI:** 10.1016/j.dib.2019.104214

**Published:** 2019-07-01

**Authors:** Fatin Izzati Minhat, Mohd Lokman Husain, Abdullah Sulaiman

**Affiliations:** a School of Marine and Environmental Sciences (PPSMS), Universiti Malaysia Terengganu, 21030 Kuala Nerus, Terengganu, Malaysia; b Institute of Oceanography and Environment (INOS), Universiti Malaysia Terengganu, 21030 Kuala Nerus, Terengganu, Malaysia; c Mineral and Geoscience Department of Malaysia, Jalan Sultan Azlan Shah, Peti Surat 1015, 30820, Ipoh, Perak, Malaysia

**Keywords:** Tropical waters, Palaeoceanography, Palaeoclimate, Micropalaeontology

## Abstract

The data presented herein were collected from the Straits of Malacca, along the west coast of Peninsular Malaysia. A 3.9 m core sample was retrieved from the Straits of Malacca in 2001. This core was continuously sub-sampled at 5-cm intervals between selected core depths of 220 cm and 380 cm. The 32 sub-samples obtained were analysed to understand the species composition of benthic Foraminifera in them and the changes in lithology during the Holocene. The data available in this article include the raw counts of different species of Foraminifera and the weight percentages of sediment of different grain sizes and organic matter at different depth. In addition, the estimated ages of the sediment samples are also provided. The chronostratigraphic framework of the core was based on radiocarbon-14 Accelerator Mass Spectrometry (AMS) dates estimated from three selected sediment intervals. The results of carbon dating were calibrated to calendar years (cal BC/AD) and calibrated radiocarbon years (cal BP). Calibration was done using the INTCAL program with a Delta R value of −19 ± 70.

**Table:** Specification table

Subject area	*Geology*
More specific subject area	*Micropalaeontology*
Type of data	*Table and graph*
How data were acquired	*Microscope, mechanical shaker with sieves (4-0.*063 mm*), loss-on-ignition (LOI) method, radiocarbon-14 Accelerator Mass Spectrometry (AMS) method, laser diffractometer particle size analyser*
Data format	*Raw and analysed*
Experimental factors	*The foraminifera data presented are raw counts data. The organic matter and sediment grain size data has been changed to percentages.*
Experimental features	*Foraminifera specimen were hand pick from each sample. Sediment grain size analysis was carried out using dry sieving technique. For radiometric age analysis all samples were subject to an acid etching pre-treatment. Organic matter were determined using the loss-on-ignition method.*
Data source location	*Waters of Kedah state, Malaysia (5.78157°N, 96.570271°E)*
Data accessibility	*All data are provided within this article*

**Table:** 

**Value of the data** • The data in this article provide the overview of the species composition and distribution of benthic Foraminifera during the Holocene from cores collected at the Straits of Malacca, Malaysia. • The raw count data for different foraminifera species provided could support future research of reconstructing palaeoceanography or palaeoclimate changes. • The sediment grain size and organic matter data provide insights into the possible depositional environment in the Straits of Malacca during the Holocene. • The radiocarbon age data provide the precise estimated ages of the sediment intervals examined. These data could be used in the development of models to interpret the Holocene environment.

## 1 Data

The 32 selected sediment sub-samples that were taken from a 3.9 m sediment core provided insight into the distribution and species composition of benthic Foraminifera over time. The raw counts of specimens picked out of the samples and benthic foraminifera species identification data are provided for selected core intervals (220–380 cm) (Table 1). Additionally, data on sediment characteristics and organic matter content are provided as weight percentages throughout the selected core intervals (Table 2). The chronological framework data are available in this article together with information on the sampled materials used for age estimation and the sample depths in the core (cm) for which the age estimates were made, with ages presented in terms of the samples’ conventional Accelerator Mass Spectrometry (AMS) age and their age in calibrated radiocarbon years (Table 3).

**Table 1:** List of identified species of benthic Foraminifera and raw count data for each species from core intervals between 220 cm and 310 cm depth in the sediment core studied from the Straits of Malacca, Malaysia. The highlighted columns indicate the absence of any foraminifera species in these core intervals.

Benthic foraminifera taxon	Core interval (cm)																	
	220–225	225–230	230–235	235–240	240–245	245–250	250–255	255–260	260–265	265–270	270–275	275–280	280–285	285–290	290–295	295–300	300–305	305–310
*Textularia* cf*. T. truncata*	0	0	0	0	0	0	0	0	0	0	0	0	0	0	0	0	0	0
*Ammonia tepida*	1	1	7	0	4	10	6	16	11	8	0	0	0	3	0	0	0	0
*Asterorotalia milletii*	0	0	0	0	0	0	0	0	0	0	0	0	0	0	0	0	0	0
*Asterorotalia* ex. var. *pulchella*	9	1	5	6	7	6	7	0	5	12	0	0	0	12	0	0	0	0
*Bolivina spathula*	0	0	0	1	1	0	1	0	0	0	0	0	0	0	0	0	0	0
*Bolivina striatula*	0	0	0	0	0	0	0	4	1	0	0	0	0	0	0	0	0	0
*Bulimina* sp. 1	0	0	0	0	0	0	0	1	0	0	0	0	0	0	0	0	0	0
*Cancris sagrum*	0	0	0	0	0	0	0	0	0	0	0	0	0	0	0	0	0	0
*Casidelina subcapitata*	0	0	0	0	0	0	0	0	0	0	0	0	0	0	0	0	0	0
*Cassidelina* cf *complanata*	0	0	0	0	1	0	0	0	0	0	0	0	0	0	0	0	0	0
*Cassidelina spinescens*	0	0	0	0	0	0	0	0	0	0	0	0	0	0	0	0	0	0
*Cymbaloporetta* cf. *C bradyi*	0	0	0	0	0	0	0	0	0	0	0	0	0	0	0	0	0	0
*Discorbinella bertheloti*	0	0	0	0	0	0	0	0	0	0	0	0	0	0	0	0	0	0
*Elphidium advenum*	0	0	1	1	2	3	0	0	1	2	0	0	0	0	0	0	0	0
*Elphidium excavatum*	0	0	0	0	0	0	1	0	0	0	0	0	0	0	0	0	0	0
*Fissurina bispinata*	0	0	0	0	0	2	0	0	0	2	0	0	0	0	0	0	0	0
*Fursenkoina* sp. 1	0	0	0	0	0	0	1	0	0	0	0	0	0	0	0	0	0	0
*Gavelinopsis praegeri*	0	0	0	0	0	0	0	0	0	0	0	0	0	0	0	0	0	0
*Hopkinsinella gabra*	0	0	0	0	0	0	0	0	0	0	0	0	0	0	0	0	0	0
*Lagena dorbignyi*	0	0	0	0	0	0	1	0	0	0	0	0	0	0	0	0	0	0
*Lagena tokiokai*	0	0	0	0	0	0	0	0	0	0	0	0	0	0	0	0	0	0
*Nonion subturgidum*	1	1	1	4	8	8	11	2	7	8	0	0	0	3	0	0	0	0
*Nonionella* cf. *N. miocenica*	0	0	0	0	0	0	0	0	2	0	0	0	0	0	0	0	0	0
*Nonionoides* sp. 1	0	0	0	0	0	0	0	0	0	0	0	0	0	0	0	0	0	0
*Pararotalia ozawai*	1	0	0	0	0	1	2	0	0	0	0	0	0	0	0	0	0	0
*Porosononion simplex*	3	1	0	0	8	0	5	0	1	3	0	0	0	0	0	0	0	0
*Procerolagena oceanica*	0	0	0	0	1	0	0	0	0	1	0	0	0	0	0	0	0	0
*Pseudononion* sp. 1	1	0	0	0	3	2	1	0	0	0	0	0	0	3	0	0	0	0
*Pseudorotalia indopacifica*	0	1	0	0	0	0	2	0	1	0	0	0	0	0	0	0	0	0
*Pseudorotalia schroeteriana*	0	0	0	0	0	0	0	0	0	0	0	0	0	0	0	0	0	0
*Sagrina jugosa*	0	0	0	0	0	0	0	0	0	0	0	0	0	0	0	0	0	0
TOTAL COUNT	16	5	14	12	35	32	38	23	29	36	0	0	0	21	0	0	0	0

Benthic foraminifera taxon	Core length (cm)													
	310–315	315–320	320–325	325–330	330–335	335–340	340–345	345–350	350–355	355–360	360–365	365–370	370–375	375–380
*Textularia* cf*. T. truncata*	0	0	0	0	0	0	0	0	0	0	0	0	1	0
*Ammonia tepida*	3	0	0	3	0	1	0	0	0	2	5	11	15	11
*Asterorotalia milletii*	0	0	0	0	0	0	0	0	0	0	2	2	12	4
*Asterorotalia* ex. var. *pulchella*	6	37	12	21	0	17	10	13	3	21	0	73	99	101
*Bolivina spathula*	0	0	0	0	0	0	0	0	0	0	0	0	4	3
*Bolivina striatula*	0	0	0	0	0	0	0	0	0	0	0	0	0	1
*Bulimina* sp. 1	0	0	0	0	0	0	0	0	0	0	0	0	0	0
*Cancris sagrum*	0	0	0	0	0	0	0	0	0	0	0	0	1	0
*Casidelina subcapitata*	0	0	0	0	0	0	0	0	0	0	0	0	0	2
*Cassidelina* cf. *C. complanata*	0	0	0	0	0	0	0	0	0	0	0	0	0	0
*Cassidelina spinescens*	0	0	0	0	0	0	0	0	0	0	0	0	1	0
*Cymbaloporetta* cf*. C. bradyi*	0	0	0	0	0	0	0	0	0	0	0	0	1	0
*Discorbinella bertheloti*	0	0	0	0	0	0	0	0	0	0	0	0	0	2
*Elphidium advenum*	0	0	0	3	0	3	3	0	0	0	1	1	3	3
*Elphidium excavatum*	3	6	1	0	0	0	0	0	0	0	0	10	8	11
*Fissurina bispinata*	1	0	0	0	0	0	0	0	0	1	2	0	0	0
*Fursenkoina* sp. 1	0	0	0	0	0	1	0	0	0	0	0	1	3	0
*Gavelinopsis praegeri*	0	0	0	0	0	0	0	0	0	0	1	1	0	0
*Hopkinsinella gabra*	0	0	0	0	0	0	0	0	0	0	0	0	1	0
*Lagena dorbignyi*	0	0	0	1	0	0	0	0	0	0	0	0	0	0
*Lagena tokiokai*	0	0	0	0	0	0	0	0	0	0	0	0	0	1
*Nonion subturgidum*	3	0	0	6	0	4	3	0	0	3	1	15	12	18
*Nonionella* cf. *N*. *miocenica*	0	0	0	0	0	0	0	0	0	0	0	0	0	3
*Nonionoides* sp. 1	0	0	0	0	0	0	0	0	0	0	0	0	0	2
*Pararotalia ozawai*	0	1	0	1	0	0	0	0	0	1	0	10	0	21
*Porosononion simplex*	0	5	1	0	0	2	3	0	0	6	3	10	5	4
*Procerolagena oceanica*	0	0	0	0	0	0	0	0	0	0	0	0	0	0
*Pseudononion* sp. 1	0	2	0	0	0	0	0	0	0	1	3	0	0	0
*Pseudorotalia indopacifica*	0	0	1	0	0	1	1	0	0	1	0	6	3	5
*Pseudorotalia schroeteriana*	0	1	0	0	0	0	0	2	0	0	0	0	0	0
*Sagrina jugosa*	0	0	0	0	0	0	0	0	0	0	0	0	1	0
TOTAL	16	52	15	35	0	29	20	15	3	36	18	140	170	192

**Table 2:** The weight percentage (%) of the sediment in each core interval (cm) composed of organic matter, sand (>63 μm), silt (4–63 μm), and clay (<4 μm). Data are from a sediment core collected from the Straits of Malacca, Malaysia.

Core interval (cm)	Organic matter (g)	Weight percentage (%)		
		Sand (>63 μm)	Silt (4–63 μm)	Clay (<4 μm)
220–225	0.82	52.5	40.6	6.9
225–230	0.32	59.7	34.7	5.6
230–235	0.17	63.4	31.4	5.2
235–240	0.14	44.1	48.3	7.6
240–245	0.16	56.1	37.6	6.3
245–250	0.10	57.0	36.6	6.4
250–255	0.11	49.6	43.0	7.4
255–260	0.28	60.0	34.0	6.0
260–265	0.25	51.2	41.4	7.4
265–270	0.29	54.9	38.4	6.7
270–275	0.30	53.4	39.8	6.8
275–280	0.31	53.1	40.1	6.8
280–285	0.31	49.0	42.4	8.6
285–290	0.31	52.5	39.1	8.4
290–295	0.28	56.9	35.5	7.6
295–300	0.27	58.5	34.8	6.7
300–305	0.27	53.5	39.0	7.5
305–310	0.28	51.1	40.7	8.2
310–315	0.30	38.6	51.0	10.4
315–320	0.30	51.5	40.0	8.5
320–325	0.23	60.9	33.0	6.1
325–330	0.25	38.2	53.1	8.7
330–335	0.26	53.0	39.5	7.5
335–340	0.26	22.9	64.1	13.0
340–345	0.25	31.0	55.2	13.8
345–350	0.30	31.1	56.7	12.2
350–355	0.31	52.3	41.0	6.7
355–360	0.30	33.6	55.5	10.9
360–365	0.29	46.1	44.6	9.3
365–370	0.31	48.3	43.8	7.9
370–375	0.14	28.7	63.5	7.8
375–380	0.16	21.8	69.4	8.8

**Table 3:** The radiocarbon-14 age estimates for each sample interval in the core collected from the Straits of Malacca, Malaysia, represented as both conventional and calibrated age data.

Sample no.	Sampled materials	Sample interval in core (cm)	Conventional AMS age (BP) ± error	2 σ age range (cal BP)	Median age (cal BP)
030019/P/250	Mollusc shells	250–255	7470 ± 30	8115–7805	7960
030019/P/365	Mollusc shells	365–370	7770 ± 30	8390–8110	8250
030019/P/375	Mollusc shells	375–380	9070 ± 30	10115–9545	9830

List of identified species of benthic Foraminifera and raw count data for each species from core intervals between 310 cm and 380 cm depth in the sediment core studied from the Straits of Malacca, Malaysia. The highlighted column indicates the absence of any foraminifera species in that core interval.

## 2 Experimental design, material and methods

The sediment core was collected in 2001 using a piston corer by the Mineral and Geoscience Department of the Government of Malaysia. The core was collected in the northern part of the Straits of Malacca, within Kedah state waters (5.78157°N, 96.570271°E) at a present-day water depth of 24.9 m. After collection, the core was divided at every 1 m of its length into a total of 4 sections. Records of lithological features were made and sand samples were collected from the core in 2001. The core was kept in open storage before it was finally sub-sampled for foraminiferal analysis in 2014. A total of 32 sediment sub-samples were collected at every contiguous 5-cm interval from 220 cm to 380 cm of the core length. The total volume of each sub-sample was approximately 228 cm^3^ with half of this volume (∼114 cm^3^) used for foraminiferal analysis and the other half used for sediment and organic matter analysis. Each sub-sample was washed over 63-μm screens under tap water. The residues retained on the sieves were transferred into weighing boats and dried overnight in an oven at 60 °C. The dried foraminiferal samples were transferred into small, pre-labelled plastic bags, in which they were kept for the counting and sorting processes. The sub-samples were then split into aliquots using a dry microsplitter and transferred onto a picking tray. Sorting and counting were carried out using the counting tray, a fine artist's brush, and dissecting tweezers, with the aid of an OLYMPUS stereomicroscope. Approximately 200 individual foraminifera were randomly picked out from each sample [1]. In samples that contained less than 200 foraminifera, all specimens were picked out [1], [2]. The selected specimens were transferred to and sorted on micro-slides for identification. Identification was based on several publications from Southeast Asia and the Southeast Pacific, such as [3], [4], [5], [6]. The organic matter content in the sediments was determined by the loss-on-ignition (LOI) method [7]. Sediment samples were oven-dried at 105 °C for 12–24 h or until a constant weight was achieved. Dry sediment sample were weighed to 5 g and placed in pre-labelled crucibles. Subsequently, the sediments were burned in a muffle furnace at 550 °C for 4 h. The percentage of organic matter in the sediment was calculated using an equation based on [7]. Samples composed of >90% fine sediments were analysed for particle size determination using a laser diffractometer (Malvern Mastersizer 2000, UK). Meanwhile, more sandy samples were analysed using conventional sieving techniques. Prior to making measurements using the laser diffractometer, all samples were treated following a modified version of the method of [8]. The presence of carbonates causes aggregation in the sediment and the incomplete separation of silt and clay [9]. Therefore, carbonates were removed by adding approximately 5–6 drops of 4 M hydrochloric acid (HCl) solution. This acid treatment destroys the crystalline lattice of clay minerals and dissolves shell fragments. Subsequently, a dispersing agent, sodium hexamethaphosphate, was added to the sediment samples (∼5 g) to allow the samples to disperse. This treatment maintained the sediment in a dispersed state by removing cemented and flocculated agents [9]. The samples were allowed to sit for at least 24 h before measurements were taken. The chronostratigraphic framework of the core intervals were carried out using mollusc shells therein. The selected samples were sent to Beta Analytic Inc. (Miami, Florida, USA) for radiometric (carbon-14) dating. The standard AMS method of radiometric dating was used, and all samples were subject to an acid etching pre-treatment. All results of carbon dating were calibrated to calendar years (cal BC/AD) and to calibrated radiocarbon years (cal BP). Calibration was done using the INTCAL program with a Delta R value of −19 ± 70.

## References

[1] Culver S.J., Snedden J.W. (1996). Foraminiferal implication for the formation of the new jersey shelf sand ridges. Palaios.

[2] Culver S.J., Mallinson D.J., Corbett D.R., Leorri E., Rouf A.A., Mohd Shazili A.N., Yaacob R., Whittaker J.E., Buzas M., Parham P.R. (2012). Distribution of foraminifera in the Setiu estuary and lagoon, Terengganu, Malaysia. J. Foraminifer. Res..

[3] Loeblich A.R., Tappan H., Moore R.C. (1964). Treatise on Invertebrate Paleontology, Part C Protista 2: Sarcodina Chiefly "Thecamoebians" and Foraminifera.

[4] Loeblich A.R., Tappan H. (1994).

[5] Szarek R. (2001). Biodiversity and Biogeography of Recent Foraminiferal Assemblages in the South-Western South China Sea (Sunda Shelf).

[6] Debenay J.-P. (2013). A Guide to 1,000 Foraminifera from the Southwestern Pacific New Caledonia: IRD Editions.

[7] Heiri O., Lotter A.F., Lemcke G. (2001). Loss on ignition as a method for estimating organic and carbonate content in sediments: reproducibility and comparability of results. J. Paleolimnol..

[8] Folk R.L. (1980). Petrology of Sedimentary Rocks: Austin.

[9] Gee G.W., Or D., Dane J.H., Topp G.C. (2002). Particle-size analysis. Methods of Soil Analysis: Part 4. Physical Methods.

